# Comparing preprocessing strategies for 3D-Gene microarray data of extracellular vesicle-derived miRNAs

**DOI:** 10.1186/s12859-024-05840-4

**Published:** 2024-06-20

**Authors:** Yuto Takemoto, Daisuke Ito, Shota Komori, Yoshiyuki Kishimoto, Shinichiro Yamada, Atsushi Hashizume, Masahisa Katsuno, Masahiro Nakatochi

**Affiliations:** 1 Public Health Informatics Unit, Department of Integrated Health Sciences, Nagoya University Graduate School of Medicine, 1-1-20 Daiko-Minami, Higashi-Ku, Nagoya, 461-8673 Japan; 2 Department of Neurology, Nagoya University Graduate School of Medicine, 65 Tsurumai-Cho, Showa-Ku, Nagoya, 466-8550 Japan; 3 Department of Clinical Research Education, Nagoya University Graduate School of Medicine, 65 Tsurumai-Cho, Showa-Ku, Nagoya, 466-8550 Japan

**Keywords:** Data imputation, Extracellular vesicle, miRNA, Normalization

## Abstract

**Background:**

Extracellular vesicle-derived (EV)-miRNAs have potential to serve as biomarkers for the diagnosis of various diseases. miRNA microarrays are widely used to quantify circulating EV-miRNA levels, and the preprocessing of miRNA microarray data is critical for analytical accuracy and reliability. Thus, although microarray data have been used in various studies, the effects of preprocessing have not been studied for Toray’s 3D-Gene chip, a widely used measurement method. We aimed to evaluate batch effect, missing value imputation accuracy, and the influence of preprocessing on measured values in 18 different preprocessing pipelines for EV-miRNA microarray data from two cohorts with amyotrophic lateral sclerosis using 3D-Gene technology.

**Results:**

Eighteen different pipelines with different types and orders of missing value completion and normalization were used to preprocess the 3D-Gene microarray EV-miRNA data. Notable results were suppressed in the batch effects in all pipelines using the batch effect correction method ComBat. Furthermore, pipelines utilizing missForest for missing value imputation showed high agreement with measured values. In contrast, imputation using constant values for missing data exhibited low agreement.

**Conclusions:**

This study highlights the importance of selecting the appropriate preprocessing strategy for EV-miRNA microarray data when using 3D-Gene technology. These findings emphasize the importance of validating preprocessing approaches, particularly in the context of batch effect correction and missing value imputation, for reliably analyzing data in biomarker discovery and disease research.

**Supplementary Information:**

The online version contains supplementary material available at 10.1186/s12859-024-05840-4.

## Background

Micro RNAs (miRNAs) are small non-coding RNAs that regulate mRNA degradation through RNA interference and are involved in regulating gene expression [1–4]. Owing to their unique expression patterns in specific tissues and cell types, miRNAs demonstrate potential as biomarkers for the diagnosis and monitoring of disease progression [1, 5, 6]. miRNAs not only function intracellularly but can also be released extracellularly through small membranous structures called extracellular vesicles, which allow them to remain stable in body fluids such as blood. Blood miRNA levels, determined through minimally invasive procedures, can serve as markers to detect the presence or progression of cancers, heart disease, and other diseases [7–9]. Notably, extracellular vesicle-derived (EV)-miRNAs have been used as diagnostic biomarkers for amyotrophic lateral sclerosis (ALS) [10–14].

miRNA-seq and miRNA microarrays are commonly used to quantify circulating miRNA levels. Thermo Fisher Scientific (Affymetrix, GeneChip) [15, 16], Agilent Technologies (SurePrint G3) [17], and Toray Industries (3D-Gene) [18–23] manufacture some of the most widely used microarray products. The preprocessing of miRNA microarray data is critical for good analytical accuracy and reliability. Optimal preprocessing methods have been proposed for Affymetrix and Agilent miRNA microarray data, and were validated in several previous studies [24–29]. The preprocessing of raw data involves noise removal, missing value imputation, normalization, and batch effect correction. Batch effects are non-biological differences attributed to variability in the measurement dates, instrumentation, reagent lots, and experimenters. Batch effects can be misinterpreted when correlated with the desired results [30, 31]. Notably, the inadvertent correction of batch effects can result in the loss of biological signals [32, 33]. Furthermore, if missing values are not properly handled, the reliability of the entire dataset is compromised, and the study risks becoming biased [34, 35]. Unfortunately, the shortcut method of deleting missing values involves deleting one or more miRNAs with missing values prior to downstream analysis, which may lead to the loss of relevant data. Moreover, normalization methods can yield false distributions if they are inappropriate for the data and can erase true biologically driven signals in downstream analyses and generate false signals [36–38].

**Fig. 1:**
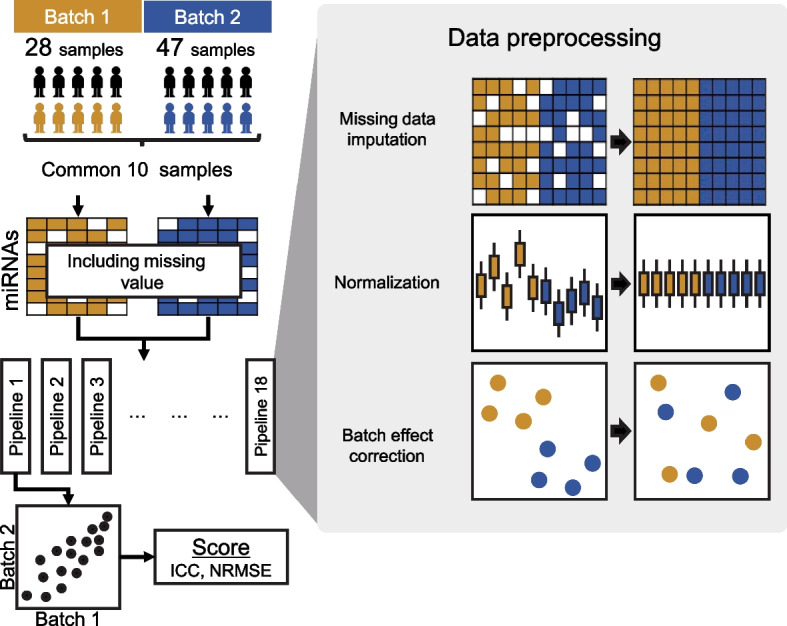
Study design

Although many studies have reported the use of Toray 3D-Gene technology for miRNA microarray analysis as a strategy for biomarker discovery and pathological studies [18–20, 39], no studies have been conducted on appropriate preprocessing methods for this method. Therefore, it is necessary to compare the results obtained using a pipeline that combines various preprocessing methods. In this study, we constructed a preprocessing pipeline for EV-miRNA data using Toray 3D-Gene technology and compared the accuracy of batch effect correction and batch-to-batch data agreement (Fig. 1).

## Methods

### Study design

Venous blood samples were collected from 28 and 47 patients from the first and second cohorts, respectively, to isolate EVs and perform miRNA microarray analyses. The first and second cohorts shared 10 common patients, for whom we evaluated data agreement and batch effects to compare the preprocessing pipelines (Fig. 1).

### Patients

We recruited subjects who were clinically diagnosed with definite, probable, clinically probable-laboratory-supported, or possible ALS based on the revised El Escorial criteria and who had no family history of ALS. Subjects with severe complications such as malignancy, heart failure, or renal failure were excluded from the study (Table 1). Patients with sporadic ALS were assessed during hospitalization. Disease onset was defined as the time at which the participant experienced weakness in any body part. All study participants were Japanese and were observed at Nagoya University Hospital between June 2014 and September 2022.

**Table 1:** Sample information for two sample batches

Parameters	Batch 1	Batch 2	Common samples
Sample number	28	47	10
Age [mean (SD)]	66.3 (8.4)	65.0 (10.8)	66.8 (5.9)
Sample number (%)			
Female	11 (39.3)	16 (34.0)	5 (50.0)
Male	17 (60.7)	31 (66.0)	5 (50.0)
Total measured miRNAs	2632	2632	2632

### Study approval

This study was conducted according to the Declaration of Helsinki and the Ethical Guidelines for Medical and Health Research Involving Human Subjects endorsed by the Japanese government. It was approved by the Ethics Review Committee of Nagoya University Graduate School of Medicine (Nos. 2013-0035 and 2015-0041), and all participants gave written informed consent before participation.

### Sample collection

Venous blood samples were collected in the supine position from patients with ALS after > 12 h of fasting and immediately after waking up during hospitalization. Serum samples were centrifuged at 1330×*g* for 10 min at 4 °C and stored at  − 80 °C until processing by NanoSomiX Japan, Inc. (Tokyo, Japan).

### Isolation of extracellular vesicles

The following processes were performed for each batch. EV fractions were isolated from the serum samples at NanoSomiX Japan using polymer-based precipitation methods [40–42]. Briefly, frozen serum samples were thawed and centrifuged at 1500×*g* for 5 min at 4 °C to remove cell debris and other contaminants; 500 µL of precleared serum was mixed with the same amount of phosphate buffered saline (PBS) and 250 µL of 30% (w/v) polyethylene glycol (molecular weight: 8000 Da; Research International, Mount Prospect, IL, USA) and incubated for 1 h at 4 °C. Crude EV fractions were precipitated by centrifugation at 1500×*g* for 30 min at 4 °C. Each EV pellet was resuspended in PBS containing 0.5% bovine serum albumin, protease inhibitors, and a phosphatase inhibitor cocktail (Thermo Fisher Scientific, Waltham, MA, USA). Isolated EVs were cryopreserved at  − 80 °C until further processing by Toray Industries, Inc. (Kamakura, Japan).

### Measurement of microarray data

Total RNA was extracted using 3D-Gene RNA Extraction Reagent with EVs isolated from the sera of subjects with ALS (Toray). The extracted total RNA was detected using a Bioanalyzer (Agilent, Santa Clara, CA, USA) and labeled using a 3D-Gene miRNA labeling kit (Toray). Half of the labeled RNA was hybridized onto a 3D-Gene (Human) miRNA Oligo chip (Toray), which was designed to detect 2632 miRNA sequences registered in the miRBase miRNA database (https://www.mirbase.org/) [43]. After washing, fluorescent signals were scanned using a 3D-Gene Scanner (Toray) and analyzed using 3D-Gene Extraction software (Toray).

**Fig. 2:**
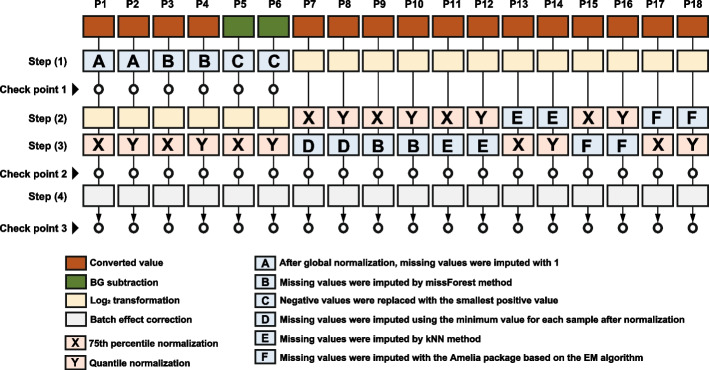
Overview of the 18 preprocessing pipelines

The following criteria were used for quality control of the microarray data. First, only miRNAs with “NG” in the Flag column in the raw data of each sample were converted to missing values. This process enables preprocessing with only reliable signals measured by reliable spot imaging. Next, background (BG) subtraction was performed by taking the mean value of the BLANK (DNA not detected) portion as the BG value and subtracting the BG value for all measurements greater than (BG value (mean) + 2SD). Measurements below (BG value (mean) + 2SD) were defined as missing values [44]. The BG subtraction value, in which NA was substituted for the missing value, was used as the converted value (Supplementary Fig. S1). The missing rate was calculated for each miRNA. miRNAs with a missing rate > 0.95 were excluded from the BG subtraction and converted value data. Finally, 75 samples containing data for 1928 miRNAs were subjected to preprocessing, as shown in the preprocessing pipeline schematic below (Fig. 2).

### Preprocessing pipeline

The details of the 18 studied pipelines are shown in Fig. 2.

#### Pipeline 1

Using the converted value (1) in each sample, the median of all the miRNA converted values was calculated, and each converted value was adjusted as follows so that the median was 25 (global normalization): each converted value/median × 25. Missing values were imputed with 1 (imputation A) [45]. (2) After imputation A, the data were log-transformed (base = 2). (3) The 75th percentile of the log-transformed data was calculated and subtracted from each data point (75th percentile normalization) [26]. (4) Batch effect correction was performed using the ComBat function in the sva R package (ver. 3.48.0) [46]. ComBat uses an empirical Bayes method to correct for batch effects, thereby adjusting for systematic variation between different experimental conditions and batches. This method specifically minimizes differences in means and heterogeneity between variables across batches, thereby reducing bias and increasing data uniformity [47].

#### Pipeline 2

Using the converted value, steps (1) and (2) proceeded as in pipeline 1. (3) Quantile normalization was performed on the log-transformed data. This normalization method sorts the values for each sample, averages the sorted values across the samples of each rank, and assigns these averages to the original order of each sample [26]. (4) Batch effect correction was performed using the ComBat function in the sva package in R (ver. 3.48.0).

#### Pipeline 3

Using the converted value, (1) missing values were imputed using the missForest package (ver. 1.5) (imputation B). missForest uses a random forest approach to predict missing values. It constructs a random forest using available data and predicts missing values based on the observed portion of the sample with missing entries. This approach allows for the imputation of missing values and maintains the integrity of the data for analysis [48]. Steps (2)–(4) were performed in the same manner as in pipeline 1.

#### Pipeline 4

Using the converted value, (1) missing values were imputed using the missForest method (imputation B). Subsequent steps (2)–(4) were performed in the same manner as in pipeline 2.

#### Pipeline 5

Using the BG subtraction value, (1) negative values were replaced with the smallest positive value (replacement C) [49]. Steps (2)–(4) were performed in the same manner as in pipeline 1.

#### Pipeline 6

Using the BG subtraction value, (1) negative values were replaced with the smallest positive value (replacement C). Steps (2)–(4) were performed in the same manner as in pipeline 2.

#### Pipeline 7

Using the converted values, we obtained (1) logarithmically transformed values (base = 2) and performed (2) 75th percentile normalization. (3) The minimum value was calculated for each sample using the normalized data and imputed as the missing values (imputation D). Finally, we performed (4) batch effect correction.

#### Pipeline 8

Using the converted values, we obtained (1) logarithmically transformed values (base = 2) and performed (2) quantile normalization. (3) Missing values were imputed with the minimum value after step (2). Finally, we performed (4) batch effect correction.

#### Pipeline 9

Using the converted value, steps (1) and (2) proceeded as in pipeline 7. We then performed (3) imputation B and (4) batch effect correction.

#### Pipeline 10

Using the converted value, steps (1) and (2) proceeded as in pipeline 8. We then performed (3) imputation B and (4) batch effect correction.

#### Pipeline 11

Using the converted value, steps (1) and (2) were performed as in pipeline 7. In step (3), missing values were imputed following the *k*-nearest neighbor (kNN) method using the impute package (ver. 1.46.0) (imputation E) [50, 51]. This imputation method is used to fill in missing values in a dataset; missing values are estimated using the values of the neighboring data points that are closest to the data point with the missing values. In this study missing values were calculated with the number of neighbors = 10 and maximum percent missing data = 0.95. We then performed batch effect correction (4).

#### Pipeline 12

Using the converted value, steps (1) and (2) were performed as in pipeline 8. In step (3), imputation E was performed. We then performed batch effect correction (4).

#### Pipeline 13

Using the converted value, (1) logarithmically transformed values (base = 2). (2) imputation E was performed. (3) 75th percentile normalization. Finally, we performed (4) batch effect correction. 

#### Pipeline 14

Using the converted value, (3) quantile normalization was performed. Steps (1﻿)–(2), (4) were performed in the same manner as in pipeline 13.

#### Pipeline 15

Using the converted value, steps (1) and (2) were performed as in pipeline 7. (3) Missing values were then imputed based on the Expectation–Maximization (EM) algorithm using the Amelia package (ver. 1.6.2) (imputation F) [52, 53]. The EM algorithm is an iterative algorithm that is widely used to complete data with missing values. In this study, m = 1. First, the initial estimate is used to complete the missing values, and the estimate is subsequently updated based on the observed values. Specifically, the E step uses the observed values to compute an estimate of the missing values, and the M step uses the estimate to update the statistics of the observed values. This process is repeated until the estimates converge. We then performed batch effect correction (4).

#### Pipeline 16

Using the converted value, steps (1) and (2) were performed as in pipeline 8. We then performed (3) imputation F. We then performed batch effect correction (4).

#### Pipeline 17

Using the converted value, (2) imputation F was performed. Steps (1), (3)﻿–(4) were performed sin the same manner as in pipeline 13.

#### Pipeline 18

Using the converted value, (2) imputation F was performed. Steps (1), (3)﻿–(4) were performed in the same manner as in pipeline 14.

### Evaluation of data agreement

Data agreement was assessed for miRNAs extracted from the 10 samples which were common to both data cohorts. The data was preprocessed in each pipeline using two extraction methods; the first method extracted only miRNAs with missing values stored in either batch, whereas the second extracted only detectable miRNAs (Supplementary Fig. S2).

The intraclass correlation coefficient (ICC) and 95% confidence intervals for the ICC were calculated using the icc function in the irr R package (ver. 0.84.1). The ICC is a statistical index used to assess the consistency and reliability of measurements of subjects when there are multiple raters or across multiple trials [54]. In this study, ‘raters’ refers to different batches, and ‘subjects’ refers to different combinations of common samples and miRNAs. ICC (2, 1) values were calculated by considering both batch 1 and batch 2 as random models. *i* is the pair number of the 10 common sample pairs between the two batches and *n*_*i*_ is the number of miRNAs evaluated for pair *i*. Hence, the ICC (2, 1) was calculated based on the $\sum_{i=1}^{10}{n}_{i}$ normalized values of miRNAs, and is defined as shown in the following equation.

$$ICC\left(\text{2,1}\right)=\frac{{MS}_{C}-M{S}_{E}}{M{S}_{C}+M{S}_{E}+\frac{2}{\sum_{i=1}^{10}{n}_{i}}\left(M{S}_{B}-M{S}_{E}\right)},$$

where the *MS*_*C*_, *MS*_*B*_, and *MS*_*E*_ are the mean squares (MSs) associated with combined common samples and miRNAs, batches, and residuals, respectively, in the analysis of variance framework. We followed the interpretation criterion which states that values near 1 and 0 indicate high and low agreement, respectively [54]. The root mean square error (RMSE) was calculated as $RMSE= \sqrt{\frac{\sum_{i=1}^{10}\sum_{j=1}^{{n}_{i}}{\left({B1}_{ij}-{B2}_{ij}\right)}^{2}}{\sum_{i=1}^{10}{n}_{i}}}$*,* where *j* is each miRNA for sample pair *i*, and *B1*_*ij*_ and *B2*_*ij*_ are the (log) normalized values of miRNA* j* for batches 1 and 2 in sample pair *i*, respectively. The RMSE was normalized by dividing it by the range (maximum–minimum) of the values for batch 1 with each pipeline as follows: $NRMSE= \frac{RMSE}{\text{max}\left({B1}_{ij}\right)-\text{min}({B1}_{ij})}$.

### Statistical analysis

The batch effect evaluation scores were calculated using a *t*-test with the first and second principal component scores obtained from principal component analysis (PCA). The hypotheses of the test with ICC were as follows: H_0_: ICC = 0, H_1_: ICC > 0. The *p*-values were corrected using the Benjamini–Hochberg method [55]. Statistical analyses were performed using R version 4.3.1 (R Foundation for Statistical Computing, http://www.R-project.org). Statistical significance was set at *p* < 0.05.

## Results

### Evaluation of batch effect correction

The converted value and BG subtraction distributions were first visualized to understand the microarray distributions of each sample. The distribution of each sample was skewed and thus, not considered normal (Supplementary Fig. S3a and b). Furthermore, the distributions of common samples (same color pairs) were different despite reflecting the same sample. Therefore, both the converted and BG subtraction values required preprocessing. Importantly, the importance of the comprehensive assessment was highlighted in this study.

**Fig. 3:**
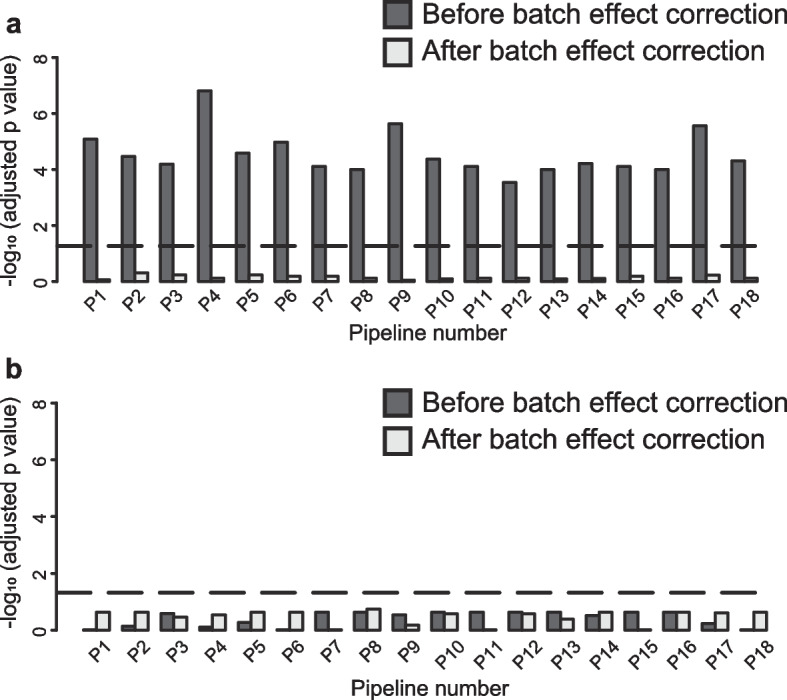
Batch effect evaluation. **a** PC1 (black bar is checkpoint 2, gray bar is checkpoint 3) and **b** PC2 (black bar is checkpoint 2, gray bar is checkpoint 3). Broken lines indicate a significance level of 0.05. *p*-values were adjusted using the Benjamini–Hochberg method for multiple comparisons

The EV-miRNA microarray data preprocessing pipeline is shown in Fig. 2. To evaluate batch effects in the preprocessing pipeline, PCA was performed before and after batch effect correction with ComBat in each preprocessing pipeline, using 10 samples common to each batch (Supplementary Fig. S4, Fig. 3). To quantitatively evaluate the batch effects, we performed *t*-tests on the batch sample scores from the first and second principal components. Before batch effect correction, the first principal component exhibited significant batch-to-batch variation, whereas no significant differences were observed after batch effect correction (Fig. 3a, b), indicating that the batch effect was effectively suppressed.

### Evaluation of missing value imputation accuracy

**Fig. 4:**
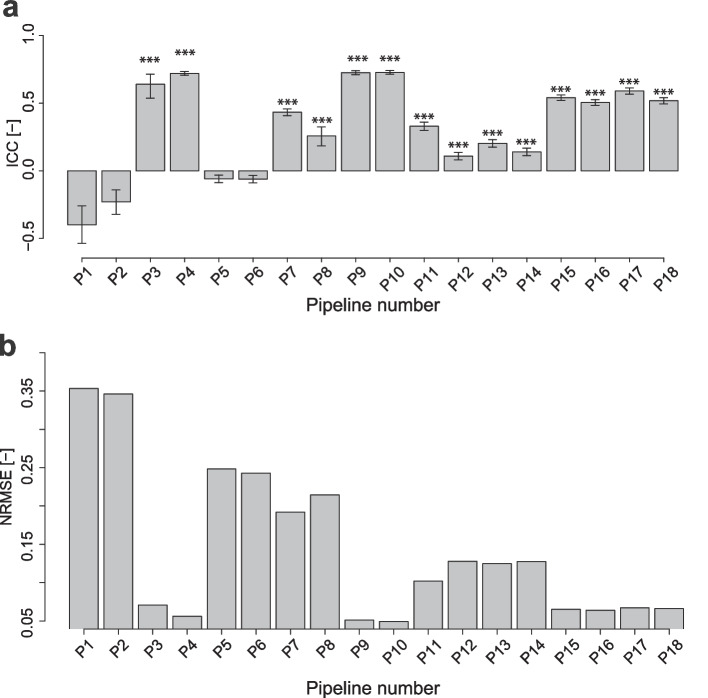
Evaluation of the missing imputation accuracy value for data with imputed values (NA rate = 0.5) at checkpoint 3 (Fig. 2). **a** Evaluation based on the ICC. Error bars represent 95% confidence intervals. Significance levels are denoted as * for adjusted *p*-values < 0.05 and *** for *p*-values < 0.001. *p*-values were adjusted using the Benjamini–Hochberg method for multiple comparisons. **b** Evaluation by NRMSE

We evaluated the agreement of imputed missing values between the two batches of 10 common samples by only considering miRNAs for which missing values were identified during measurement (Supplementary Fig. S2). Scatter plots were used to compare the measured and imputed values for the 10 samples (Supplementary Fig. S5). The accuracy of missing value imputation was evaluated using two agreement scores, i.e., the ICC and normalized RMSE (NRMSE) (Fig. 4a, b).

Pipelines 3 (ICC = 0.64, NRMSE = 0.071), 4 (ICC = 0.72, NRMSE = 0.056), 9 (ICC = 0.73, NRMSE = 0.051), and 10 (ICC = 0.73, NRMSE = 0.049) were highly consistent (Fig. 4a, b). Pipeline 10 was shown to have the best agreement, but the difference in accuracy between pipelines 4 and 10 was very small. Thus, we concluded that both were the best performing pipelines. Although pipelines 3, 4, 9, and 10 differed in normalization methods and order of processing (missing value imputation followed by normalization or normalization followed by missing value imputation), they all used the missForest missing value imputation method. Because missForest is a nonparametric imputation method that employs a random forest to learn from the observed value population, it can perform data imputation in accordance with the observed value patterns, resulting in smaller discrepancies between the imputed missing and observed values. We examined the agreement of each processing step in the pipeline after missForest processing (Fig. 2, checkpoint 1) and found that pipelines 3 and 4 exhibited moderate agreement (Supplementary Fig. S7a and b, ICC = 0.37, NRMSE = 0.090). However, slightly lower agreement was seen for pipeline 3 after normalization and batch effect correction (Fig. 2, checkpoint 2–3) (Supplementary Fig. S7c and d, Fig. 4). The scatter plots for pipeline 3 after batch effect correction showed variability between samples, particularly in sample S003 (green), which appears off-diagonal with low-signal values (Supplementary Fig. S5). Although 75th percentile normalization aims to normalize data such that the top 25% of values (75th percentile) become 1, it may not sufficiently correct for the influence of low-signal missing values (Supplementary Fig. S7c and d). In contrast, quantile normalization was performed for pipeline 4 after checkpoint 2 (ICC = 0.51, NRMSE = 0.080), which is highly effective in harmonizing distributions across datasets [56, 57]. Based on previous findings, we presumed that this method would effectively correct batch effects and improve agreement. Therefore, among the pipelines evaluated in this study, pipeline 4 was the most suitable combination and sequence for preprocessing to achieve high agreement in missing value imputation.

Pipelines using missing value imputation methods other than missForest were examined for agreement after correcting for batch effects (pipeline 1: ICC = − 0.40, NRMSE = 0.35; pipeline 2: ICC = − 0.23, NRMSE = 0.35; pipeline 5: ICC = − 0.059, NRMSE = 0.25; pipeline 6: ICC = − 0.061, NRMSE = 0.24; pipeline 7: ICC = 0.43, NRMSE = 0.19; pipeline 8: ICC = 0.26, NRMSE = 0.21; pipeline 11: ICC = 0.33, NRMSE = 0.10; pipeline 12: ICC = 0.11, NRMSE = 0.13; pipeline 13: ICC = 0.20, NRMSE = 0.13; pipeline 14: ICC = 0.14, NRMSE = 0.13; pipeline 15: ICC = 0.54, NRMSE = 0.07; pipeline 16: ICC = 0.51, NRMSE = 0.06; pipeline 17: ICC = 0.59, NRMSE = 0.07; and pipeline 18: ICC = 0.52, NRMSE = 0.07; Fig. 4a, b). These pipelines exhibited lower agreement than pipelines 3 and 4. Pipelines 1 and 2 normalized the data by ensuring that the median became 25 and then imputed a constant value of 1 for missing values. This method introduced significant differences between the imputed and measured values, resulting in low agreement. Subsequent batch effect correction had limited effects. Pipelines 7 and 8 imputed a minimum value below the BG + 2SD for missing values after normalization. Similar to pipelines 1 and 2, the introduction of a constant value resulted in marked discrepancies between the imputed and measured values, and only moderate improvements in agreement after batch effect correction. Pipelines 5 and 6 replaced missing values with the minimum BG subtraction value for measurements below BG + 2SD. Pipelines 1 and 2 imputed a constant of 1 to the missing values, and pipelines 3 and 4 replaced the missing values with the minimum background subtraction value. Therefore, for the same sample, a specific value was imputed regardless of the miRNA. This is a simple method that differs from machine learning-based imputation methods such as those used in missForest. However, this method increases discrepancies between the actual measurements and imputed values, which affected the observed values of many miRNAs. Furthermore, we demonstrated that maintaining the accuracy of missing value imputation remains crucial even when normalization and batch effect correction are applied during preprocessing. The preprocessing pipelines using kNN-based missing value completion (pipelines 11–14) all showed low agreement (pipeline 11: ICC = 0.33, NRMSE = 0.10; pipeline 12: ICC = 0.11, NRMSE = 0.13; pipeline 13: ICC = 0.20, NRMSE = 0.13; and pipeline 14: ICC = 0.14, NRMSE = 0.13; Fig. 4a,b). Therefore, the kNN approach is not suitable for the present microarray data set. In contrast, the preprocessing pipelines based on the EM algorithm (pipelines 15–18) showed moderate agreement (pipeline 15: ICC = 0.54, NRMSE = 0.07; pipeline 16: ICC = 0.51, NRMSE = 0.06; pipeline 17: ICC = 0.59, NRMSE = 0.07; and pipeline 18: ICC = 0.52, NRMSE = 0.07; Fig. 4a,b), but remained inferior to missForest.

### Influence of preprocessing on measured values

We assessed the influence of preprocessing on the 10 shared samples from the two batches using only miRNAs for which measurements were acquired (Supplementary Fig. S2). Scatter plots were used to compare the measured values for the different batches (Supplementary Fig. S6). The influence of preprocessing on measured values was evaluated using the ICC and NRMSE.

**Fig. 5:**
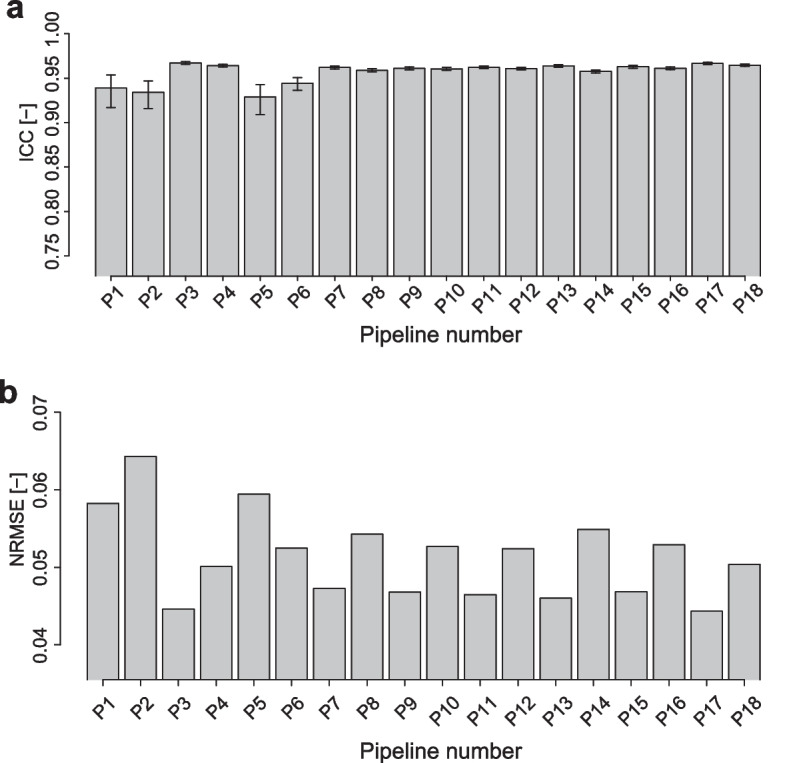
Evaluation of the influence of preprocessing on solely measured data (NA rate = 0) at checkpoint 3 (Fig. 2). **a** Evaluation based on the ICC. Error bars represent 95% confidence intervals. *p*-values were adjusted using the Benjamini–Hochberg method for multiple comparisons. All preprocessing pipelines were significant at an adjusted *p* < 0.001. The null hypothesis posits that there is no agreement between the two batches. **b** Evaluation by NRMSE

The ICC values were above 0.9 and NRMSE values were below 0.07 for all pipelines, indicating that preprocessing improved agreement and had a minimal impact on the measured values (Fig. 5a and b). With the exception of pipelines 5 and 6, the odd-numbered pipelines exhibited slightly better agreement than the even-numbered pipelines (Fig. 5a,b); specifically, pipeline 1 (ICC = 0.94, NRMSE = 0.058), pipeline 2 (ICC = 0.93, NRMSE = 0.064), pipeline 3 (ICC = 0.97, NRMSE = 0.045), pipeline 4 (ICC = 0.96, NRMSE = 0.050), pipeline 7 (ICC = 0.96, NRMSE = 0.047), pipeline 8 (ICC = 0.96, NRMSE = 0.054), pipeline 9 (ICC = 0.96, NRMSE = 0.047), pipeline 10 (ICC = 0.96, NRMSE = 0.053), pipeline 11 (ICC = 0.96, NRMSE = 0.046), pipeline 12 (ICC = 0.96, NRMSE = 0.052), pipeline 13 (ICC = 0.96, NRMSE = 0.046), pipeline 14 (ICC = 0.96, NRMSE = 0.055), pipeline 15 (ICC = 0.96, NRMSE = 0.047), pipeline 16 (ICC = 0.96, NRMSE = 0.053), pipeline 17 (ICC = 0.97, NRMSE = 0.044), and pipeline 18 (ICC = 0.96, NRMSE = 0.050). The ICC *p*-values were significant for all pipelines. The odd-numbered pipelines utilized 75th percentile normalization, whereas the even-numbered pipelines used quantile normalization. For the pipeline 5 and 6 pairs, pipeline 6 (ICC = 0.95, NRMSE = 0.052) showed slightly higher agreement than pipeline 5 (ICC = 0.94, NRMSE = 0.059).

When comparing measured values in both batches (NA rate = 0), pipelines 1, 2, 5, and 6 were found to be slightly less accurate (Fig. 5a,b). Considering the results regarding missing value imputation accuracy described in the previous section, the difference in agreement between the measured values may be related to the accuracy of missing value completion (Fig. 4a and b). Pipelines 1, 2, 5, and 6 are significantly less consistent after missing value processing, while pipelines 3 and 4 are somewhat more consistent (Supplementary Fig. S7a and b; check point 1, Supplementary Fig. S7c and d; check point 2). Therefore, pipelines 1, 2, 5, and 6 are data sets that contain incorrect imputed values, indicating that the inaccuracy remains after normalization and batch effect correction. As the data with imputed values (NA rate = 0.5) accounted for approximately 20% of the total miRNAs, incorrect imputation introduced a non-negligible bias in the results (Table 2). These results suggest that the accuracy of imputation carries over after normalization and batch effect correction, highlighting the importance of optimal preprocessing methods in the preprocessing pipeline.

**Table 2:** Number and rate of miRNAs with missing value rate = 0.5, 0 in common samples

NA rate	S001	S002	S003	S004	S005	S006	S007	S008	S009	S010	Mean	SD
0.5	445 (0.17)	577 (0.22)	766 (0.29)	412 (0.16)	367 (0.14)	467 (0.18)	483 (0.18)	413 (0.16)	616 (0.23)	543 (0.21)	508.9 (0.19)	119.5
0	919 (0.35)	781 (0.30)	760 (0.29)	1098 (0.42)	986 (0.37)	968 (0.37)	729 (0.28)	1025 (0.39)	797 (0.30)	747 (0.28)	881 (0.33)	133.6

NA rate = 0.5 indicates miRNAs missing in either batch, NA rate = 0 indicates miRNAs measured in both batches. Number of miRNAs (rate of miRNAs)

## Discussion

This study compared 18 different preprocessing pipelines for data concordance and batch effects in 10 common EV-miRNA samples derived from two cohorts using 3D-Gene technology. To date, there have been no reports comparing preprocessing methods in 3D-Gene. We found clear differences in the performance of each preprocessing pipeline, emphasizing the importance of validating a preprocessing pipeline for 3D-Gene technology. Furthermore, the results of this study provide useful insights for future analyses of the same arrays. Specifically, for each pipeline, we evaluated the missing value completion accuracy for miRNAs in which one of the miRNAs is missing from batch to batch, and the impact of preprocessing on miRNAs in which both miRNAs could be measured in each batch.

Batch effects are a common contributor to data heterogeneity between different experimental batches [30, 31]. In the present study, the impact of batch effects was clearly demonstrated by the differences in principal component scores between pipelines. Correction via ComBat improved data agreement across all pipelines. However, although correction suppressed the batch effect in all pipelines, the missing value imputation accuracy was low, particularly for pipelines 1, 2, 5, 6, 7, and 8 (Fig. 4a,b). Therefore, it is important to evaluate both batch effects and data agreement when identifying an optimal preprocessing pipeline.

We found that the method used to impute missing values had strong effects on data agreement. In particular, pipelines 3, 4, 9, and 10 using missForest had high agreement scores. missForest uses a nonparametric approach and a random forest algorithm that iteratively predicts missing values from the observed portion of the data. After each iteration, the algorithm updates the missing values with the predictions and continuously improves its accuracy until convergence or a set number of iterations is reached. Pipelines 3 and 4 utilized missForest before normalization, whereas pipelines 9 and 10 applied missForest after normalization, suggesting that missForest can accurately impute missing values both before and after normalization. However, simple strategies which assign constant values, such as those used in pipelines 1, 2, 5, 6, 7, and 8, may result in discrepancies with actual values (Fig. 4a,b). Therefore, the selection of an appropriate missing value imputation method is important for improving data agreement between batches. Moreover, this study also observed low agreement when performing missing value completion based on kNN (pipelines 11–14) and the EM algorithm (pipelines 15–18). One reason for this low agreement is that the parameters in each algorithm were not optimized. For example, the optimization of k (number of neighbors) in kNN-based missing value completion, and the optimization of the number of multiple imputations in EM algorithm-based missing value completion. kNN missing value completion in pipelines 11–14 was influenced by the fact that the data had a high missing value rate. When the missing rate is low, observations that are very similar to those with missing values are more likely and kNN imputation is more accurate. However, as the rate of missing data increases, the remaining perfect observations become less similar, leading to fewer nearest neighbor matches and less accurate input values [58]. Furthermore, when the rate of missing data is high, the risk of assigning values from observations that are not truly similar increases, which introduces bias and further reduces the accuracy of the assignments. Therefore, identifying an acceptable missing value rate may be useful in future analyses. There are few reports which apply the Amelia function to microarray data, and in this study, a single imputation was performed using the m = 1 setting in order to simply measure the effect of the EM algorithm. In future studies, by increasing the value of m, we plan to adopt an approach that uses the average from multiple imputation results to improve the accuracy of the completion. This is expected to reduce the risk of relying on a single completion result and to make data analysis more reliable.

Agilent SurePrint G3 data are commonly normalized using 75th percentile and quantile normalization methods [26]. The 75th percentile normalization method reduces the influence of outliers in the dataset by focusing on the upper quartile of data (above the 75th percentile), which is useful when the data demonstrates high variability or when expression levels are low. It is therefore less influenced by outliers in the lower quartiles, allowing for more reliable normalization in biological analyses [59]. This method is particularly suitable for the present miRNA microarray data, as the imputed values have low signal values. Quantile normalization arranges the values in the dataset in rank order and normalizes each data point such that it is in the same percentile as the original data distribution, thereby enabling the uniform distribution of data points across datasets [60]. In addition, other previous studies have identified quantile normalization as the method which most significantly reduces data variability in the case of plasma-derived miRNA microarray data using a TaqMan OpenArray Human MicroRNA Panel (Applied Biosystems, Thermo Fisher Scientific, CA) [61]. Thus, although 75th percentile normalization is suitable when dealing with outliers, quantile normalization may be useful when integrating data. Building on our evaluation of data integrity, future studies should evaluate the impact of normalization on biological differences. Furthermore, the order in which normalization and missing value completion were performed did not influence the accuracy of missing value completion. However, previous publications on proteome data have reported higher performance when normalization is performed first, followed by missing value completion [62]. Thus, the optimal pipeline differs depending on the data handled, sample origin, and other factors, suggesting the importance of the pipeline comparisons in this study.

Although our study has provided valuable insights, it is imperative to acknowledge its limitations and to identify areas requiring attention for future investigations and methodological improvement. Our analysis was narrowly concentrated on miRNA profiles derived from the serum exosomes of patients with ALS, and limited to two distinct cohorts. This specificity underlines the need for caution in generalizing our findings, as the scope was confined to a single disease (ALS) and the sample type was restricted to blood-derived exosomes. Such limitations underscore the potential for overfitting due to the insufficient number of samples within our narrowly defined cohort and sample type. It is essential to acknowledge that the outcomes from preprocessing pipelines can significantly differ across various disease cohorts, sample types, and exosome extraction methodologies. To move beyond these limitations, it is imperative to extend our research to encompass a broader spectrum of disease populations and sample types. Future efforts should aim to apply our preprocessing pipeline to conduct comparative analyses of miRNA profiles between patients with ALS and healthy controls. Such initiatives will not only aid in identifying potential ALS biomarkers but will also contribute to a deeper understanding of disease mechanisms.

In addition, we emphasize the critical role of data sharing in promoting the development of robust pipelines within the scientific community. We encourage fellow researchers to augment and leverage our publicly accessible dataset to improve methodological precision. Collective endeavors in data enrichment and sharing are vital for producing more reliable pipelines and improving the reproducibility of research findings. Leveraging these findings, future research could focus on comparative studies between ALS-derived EV-miRNA profiles and those of controls, as well as identifying outcome-associated miRNAs in patients with ALS.

Comparing the various methods would be advantageous for more appropriate pipeline construction. However, as ComBat was found to be markedly superior in the context of our study, we did not extend the analysis to include other methods. A common method for adjusting batch effects is to add batch information as a covariate during expression difference analysis. However, in this study, we could not examine this method because we did not aim to perform expression difference analysis and did not obtain the target group to evaluate differential expression. In the future, it would be beneficial to evaluate preprocessing methods that include batch effect correction when performing expression analysis. We believe that such comparisons will be useful to further refine batch effect correction strategies.

With respect to the impact on inter-group comparisons, the preprocessing pipelines in this study were evaluated in terms of missing value completion and batch effect correction, but it was not possible to evaluate whether the group differences (to be compared in biomarker identification studies) were correctly maintained. In the future, we will obtain two groups of data from multiple batches and conduct similar validation of preprocessing pipelines to evaluate the usefulness of each preprocessing pipeline for biomarker studies.

As batch effects (i.e., the frequency of missing values) and data distribution depend on the type of measured data, it is necessary to validate the suitability of preprocessing pipelines across different data types. Our approach of comparing preprocessing pipelines is important for integrated analyses using multiple cohorts, and we believe it will enable the efficient analysis of complex large-scale datasets with numerous cohorts. Furthermore, we expect that this approach will contribute to disease research and diagnostic biomarker discovery using 3D-Gene-based microarray data.

## Conclusions

Our results demonstrate the importance of preprocessing in EV-miRNA microarray data analysis. A comparative evaluation of 18 different preprocessing pipelines using 3D-Gene microarray data showed that the choice of preprocessing strategy had a significant impact on the agreement between two data batches, especially in the context of batch effect correction and missing value imputation. The batch effect correction method ComBat was able to effectively suppress batch effects in all pipelines. The pipelines employing the missForest method for data imputation showed remarkably high agreement and accuracy, indicating its suitability for EV-miRNA data analyses. In contrast, pipelines using the simpler constant-value imputation method showed lower agreement. Finally, our comparison of the type and order of preprocessing for 3D-Gene data highlighted the advantages and disadvantages of each preprocessing method and can aid the development of context-specific pipelines.

### Supplementary Information

Supplementary Material 1.

## Data Availability

The datasets generated and analyzed during the current study are available in the Gene Expression Omnibus (GEO) database at the National Center for Biotechnology Information (NCBI) and accessible through GEO series accession number GSE253541, http://www.ncbi.nlm.nih.gov/projects/geo/. The codes used to perform preprocessing are available in the Supplementary Information.

## Abbreviations

ALS: Amyotrophic lateral sclerosis
EV-miRNA: Extracellular vesicle-derived microRNA
ICC: Intraclass correlation coefficient
NRMSE: Normalized root mean square error
PBS: Phosphate buffered saline
PCA: Principal component analysis
