# Supplementary material for: Comparing preprocessing strategies for 3D-Gene microarray data of extracellular vesicle-derived miRNAs
Source: BMC Bioinformatics. 2024 Jun 20;25:221. doi: 10.1186/s12859-024-05840-4 (PMC11188187; doi:10.1186/s12859-024-05840-4)
Supplement: Supplementary file 1 — Supplementary Material 1. [file 12859_2024_5840_MOESM1_ESM.zip › 12859_2024_5840_MOESM1_ESM/Supplementary_information.docx]

Supplementary information for

**“Comparing preprocessing strategies for 3D-Gene microarray data of extracellular vesicle-derived miRNAs”**

Yuto Takemoto^1^, Daisuke Ito^2^, Shota Komori^2^, Yoshiyuki Kishimoto^2^, Shinichiro Yamada^2^, Atsushi Hashizume^2,3^, Masahisa Katsuno^2,3^, Masahiro Nakatochi^1*^

^1^Public Health Informatics Unit, Department of Integrated Health Sciences, Nagoya University Graduate School of Medicine, 1-1-20 Daiko-Minami, Higashi-ku, Nagoya 461-8673, Japan

^2^Department of Neurology, Nagoya University Graduate School of Medicine, 65 Tsurumai-cho, Showa-ku, Nagoya 466-8550, Japan

^3^Department of Clinical Research Education, Nagoya University Graduate School of Medicine, 65 Tsurumai-cho, Showa-ku, Nagoya 466-8550, Japan

## *Corresponding author

Masahiro Nakatochi, Ph.D.

Public Health Informatics Unit, Department of Integrated Health Sciences, Nagoya University Graduate School of Medicine, Nagoya 461-8673, Japan

E-mail: mnakatochi@met.nagoya-u.ac.jp;

Tel./Fax: +81-52-719-1923

## Supplementary figure 1


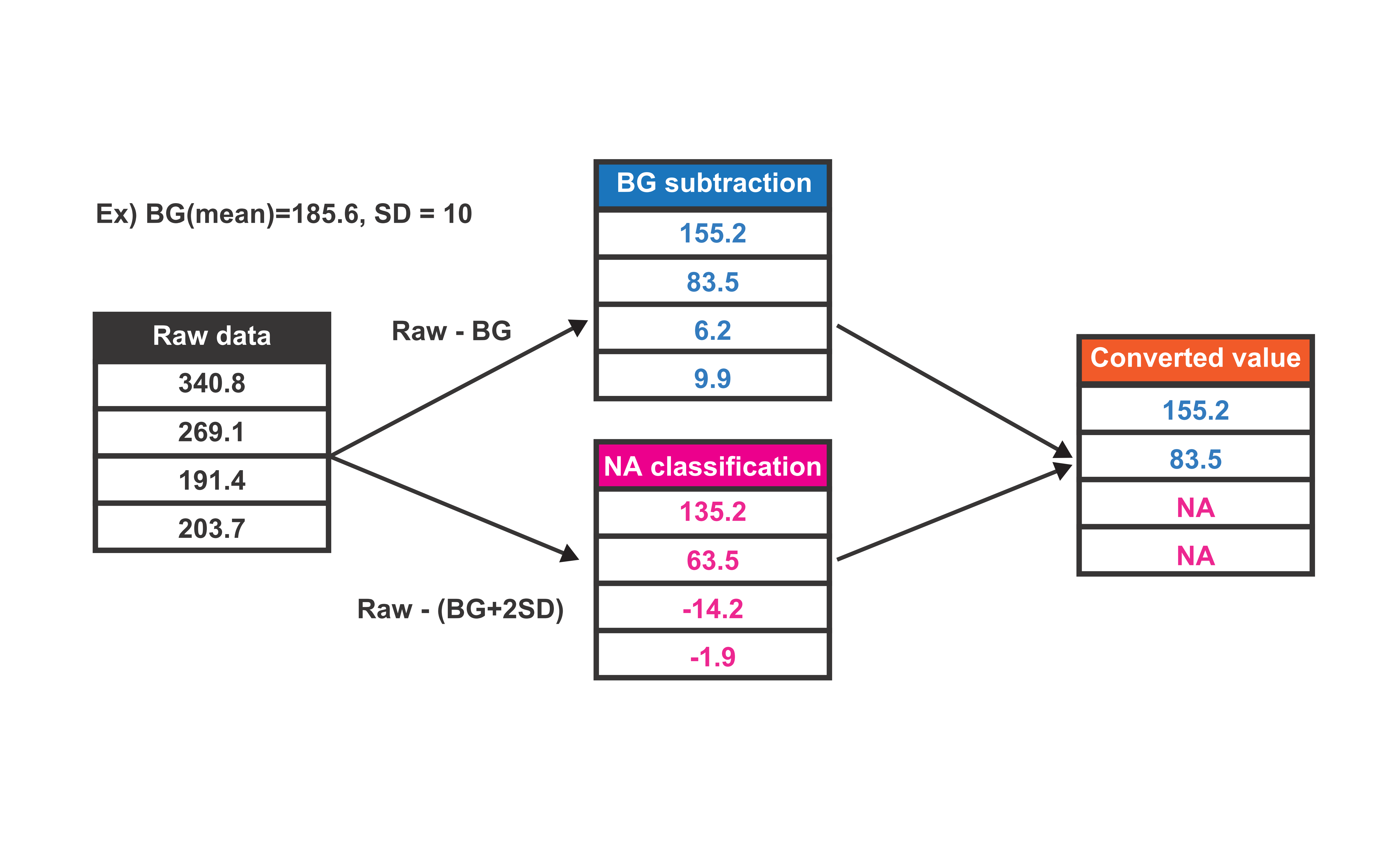


Definition of missing values in 3D gene microarray data.

## Supplementary figure 2


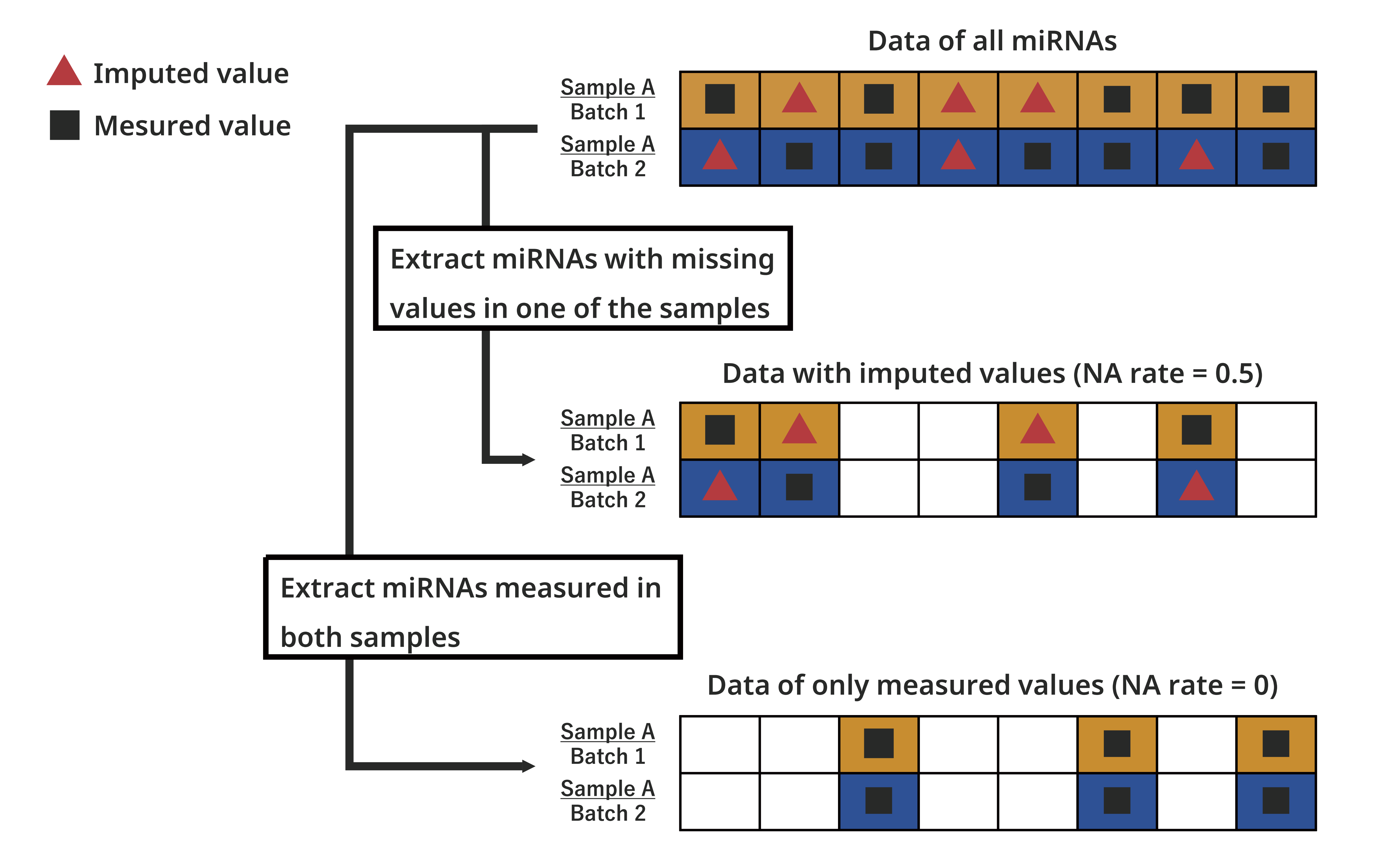


Two methods for data extraction. Red triangles indicate miRNAs whose missing values were imputed and black squares indicate miRNAs that were measured.

## Supplementary figure 3





Box-and-whisker diagram of (a) BG subtraction value and (b) converted value. Outliers are not shown. Color labels indicate the 10 common samples. If S001-S010 in batches 1 and 2 have the same number (color), they refer to samples obtained from the same patient. Samples after S011 are unique for each batch, even if they have the same number.

## Supplementary figure 4


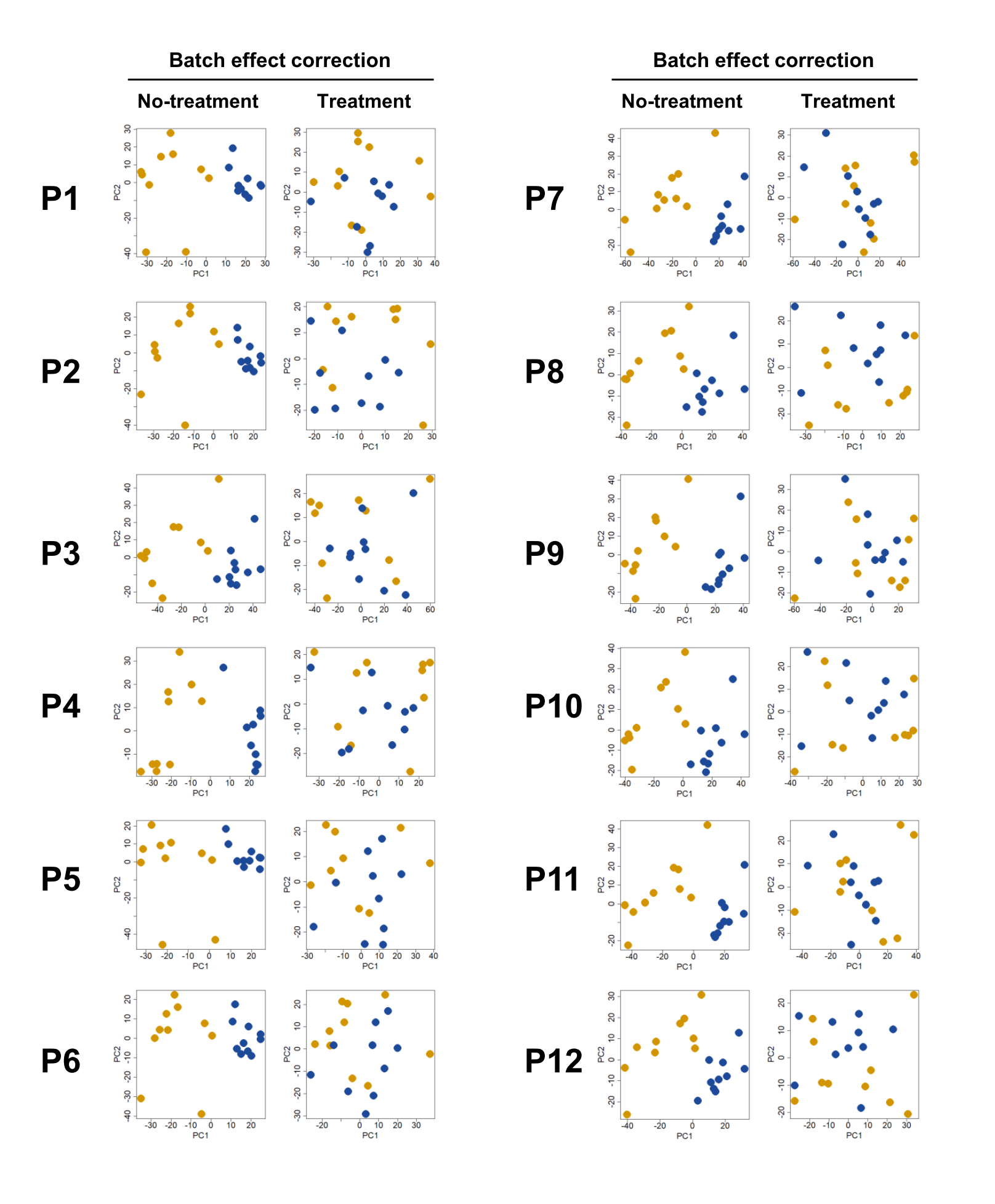


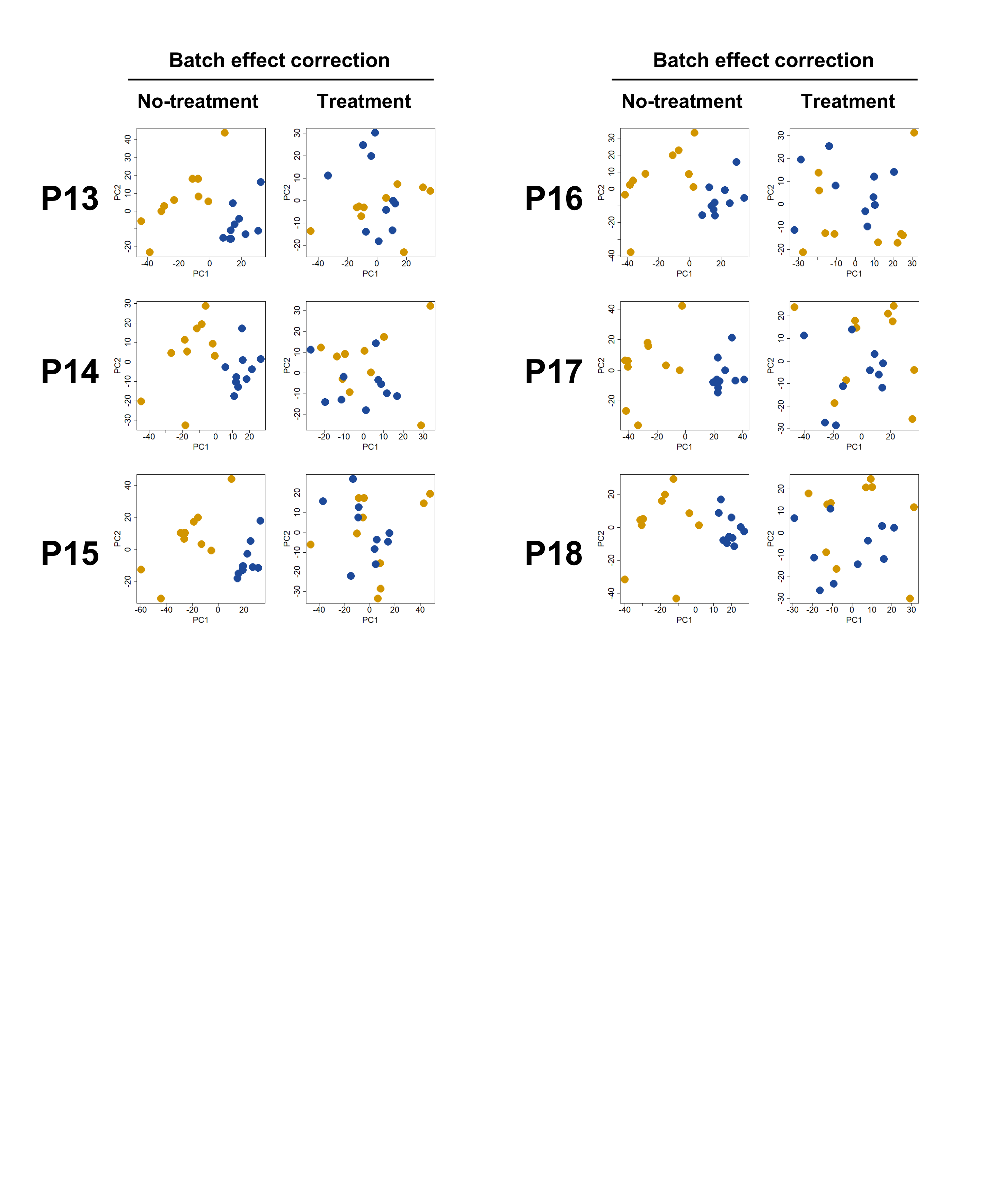


Scatter plots in PCA for batch effect evaluation. The vertical axis reflects the first principal component score and the horizontal axis reflects the second principal component score. The yellow and blue dots indicate batch 1 and batch 2, respectively.

## Supplementary figure 5





Scatter plots generated using imputed value and measured value pairs (NA rate = 0.5) for 10 common samples at checkpoint 3. Normalized values (log) for batch 1 on the horizontal axis, (log) normalized values for batch 2 on the vertical axis. Color labels indicate the 10 common samples.

## Supplementary figure 6


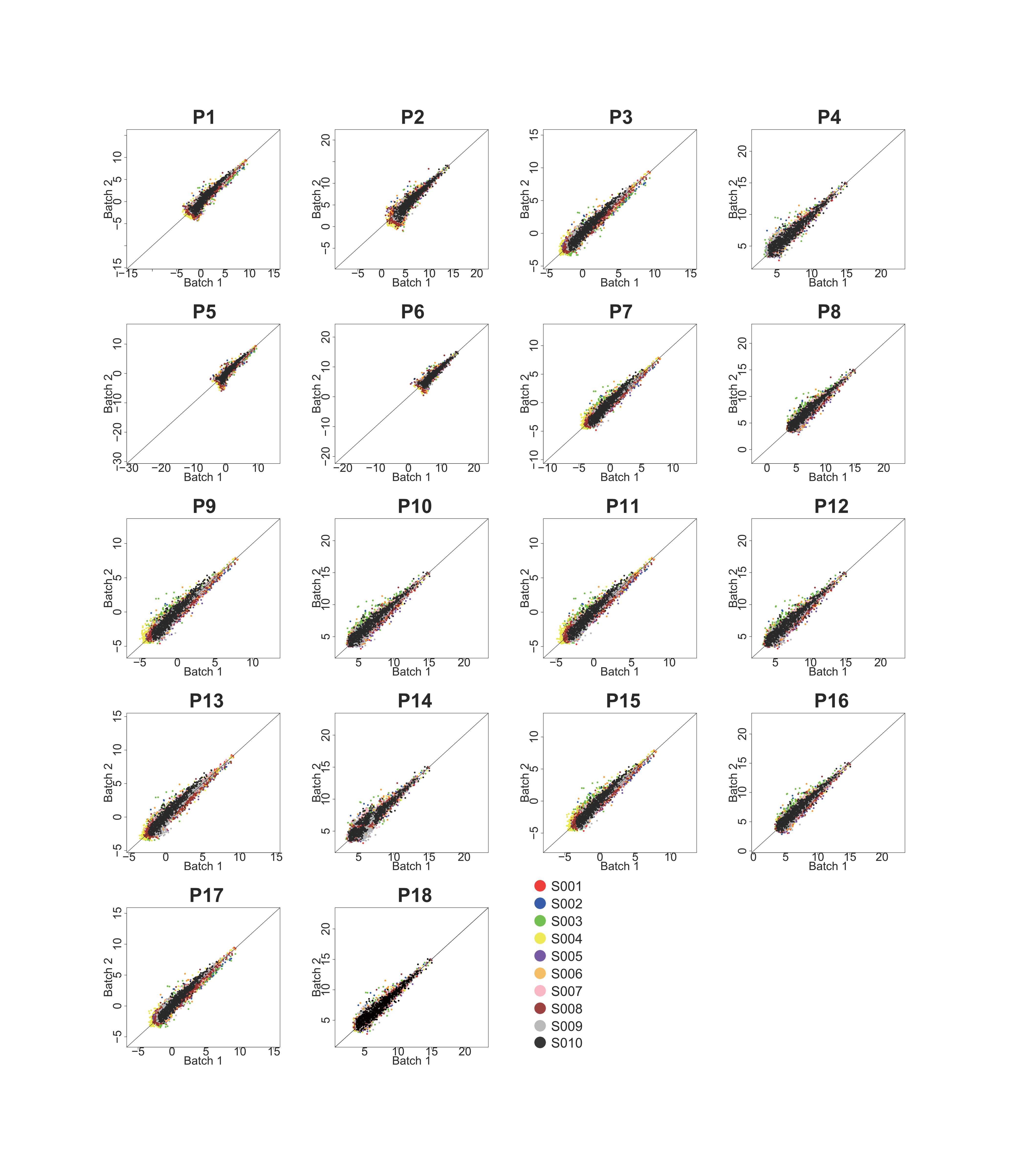


Scatter plots generated using measured values in both batches (NA rate = 0) for 10 common samples at checkpoint 3. Normalized values (log) for batch 1 on the horizontal axis, (log) normalized values for batch 2 on the vertical axis. Color labels indicate the 10 common samples.

## Supplementary figure 7


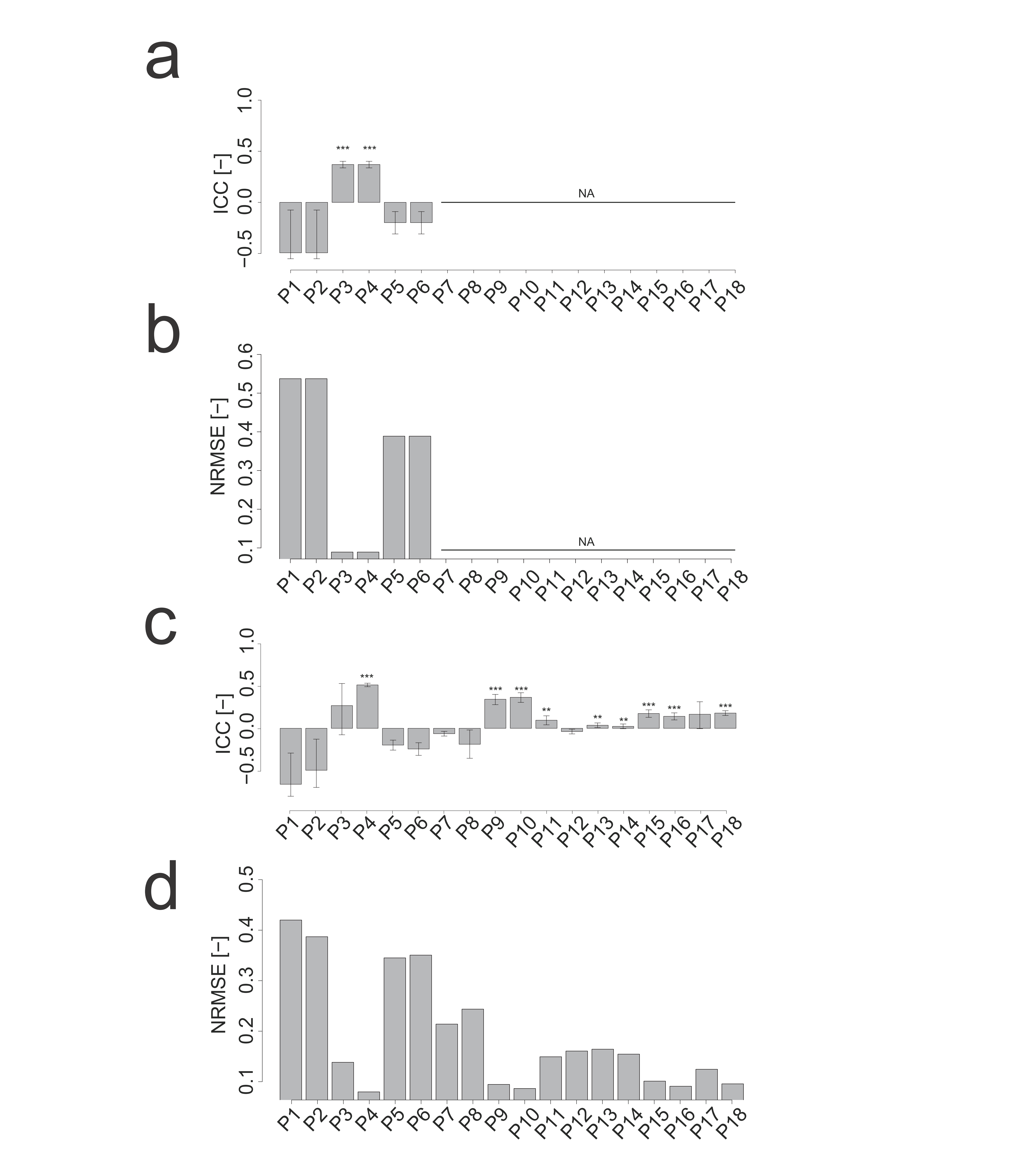


Evaluation of the missing imputation accuracy value for data with imputed values (NA rate = 0.5) (Figure 2). Evaluation based on (a) ICC and (b) NMRSE at checkpoint 1 and (c) ICC and (d) NMRSE at checkpoint 2. Error bars represent 95% confidence intervals. Significance levels are denoted as ** for adjusted *p*-values < 0.01 and *** for *p*-values < 0.001. *p*-values were adjusted using the Benjamini–Hochberg method for multiple comparisons. (b) Evaluation by NRMSE.

## Preprocessing pipeline code

R source code for 18 preprocessing pipelines.
